# Debonding and Clean-Up in Orthodontics: Evaluation of Different Techniques and Micro-Morphological Aspects of the Enamel Surface

**DOI:** 10.3390/dj8020058

**Published:** 2020-06-17

**Authors:** Maurizio D’Amario, Sara Bernardi, Daniele Di Lauro, Giuseppe Marzo, Guido Macchiarelli, Mario Capogreco

**Affiliations:** 1 Department of Life, Health and Environmental Sciences, University of L’Aquila, 67100 L’Aquila, Italy; maurizio.damario@univaq.it (M.D.); dnl.dilauro@gmail.com (D.D.L.); giuseppe.marzo@cc.univaq.it (G.M.); gmacchiarelli@univaq.it (G.M.); mario.capogreco@cc.univaq.it (M.C.); 2 Microscopy Center, University of L’Aquila, 67100 L’Aquila, Italy

**Keywords:** debonding, clean-up, SEM, magnification system, orthodontic treatment

## Abstract

There is currently no consensus on the best way to remove adhesive remnants from teeth following debonding. The main objective of this study is to evaluate and compare the effectiveness of four adhesive resin removal (clean-up) techniques, performed with or without the use of an operative microscope. Forty human teeth were duplicated using an epoxy resin for impregnation. Brackets were bonded to teeth and debonded from teeth. Then, the samples were randomly divided into two equal groups—the naked eye group and the magnification group—and further subdivided into four equal subgroups, in order to compare the different techniques used for the clean-up. Each subgroup was formed of five natural teeth with the respective pre- and post-bonding replicas. Macro- and micro-analysis by means of a stereomicroscope and scanning electron microscopy evaluated, qualitatively and quantitatively, the adhesive remnant index and the damage index of the enamel. Overall, the magnification improved the removal of resins compared to the naked eye (*p* < 0.001), and the use of magnification constantly reduced resin residual and surface damage. Enamel damage and adhesive residual from the clean-up procedures represent an ascertained risk in orthodontics. The use of a magnification system improves the quality of debonding and clean-up techniques in a significant way.

## 1. Introduction

The end of orthodontic therapy with brackets coincides with two stages—debonding and clean-up—respectively characterized by the removal of brackets and by finishing and polishing the enamel’s surface. Resin removal from the enamel’s surface after debonding is crucial and can affect the patient’s aesthetic and oral health, especially considering plaque accumulation [1,2,3]. 

Available debonding and clean-up techniques cannot completely clean the vestibular surface of teeth and can lead to temporary alterations of the morphology of the underlying enamel that are visible to the naked eye [4].

There are no established guidelines for either debonding or for clean-up [5,6,7], due to the lack of studies evaluating (by means of quantitative analysis) the enamel’s wear or residual resin remaining after various methods of bracket removal.

The aim of this in vitro study is to evaluate and compare the effectiveness of four clean-up techniques, performed with or without the use of an operative microscope. The null hypothesis tested was that the adhesive remnants and the enamel damage would not be affected by the techniques employed for the removal of adhesive resin or the use of magnification for its removal. 

## 2. Materials and Methods

### 2.1. Ethics

The study was approved by the Internal Review Board of the University of L’Aquila, n. prot. CONS_001-19 vers. 2. 27.03.2020, 10/2020. All subjects gave their informed consent for inclusion before they participated in the study.

### 2.2. Sample Preparation

A total of 40 human teeth (premolars and incisors) without caries, defects, or cracks on the crowns were selected and stored in 0.5% chloramine-T aqueous solution at 4 °C for no longer than 1 week after extraction. The teeth were extracted due to periodontal disease and a high mobility. The roots of these teeth were amputated 2 mm apically at the buccal cement–enamel junction. Coronal parts were polished with pumice and a brush mounted on a contra-angle hand piece.

### 2.3. Duplication Protocol

A duplication flask was realized, comprised of a vessel consisting of a base on which crowns were splinted with wax and with walls high enough to accommodate the duplicating silicone. Then, the fast duplicating addition polyvinylsiloxane (Elite Double 22 Fast, Zhermack Spa, Badia Polesine, Italy) was mixed manually, according to the manufacturer’s instructions. Once mixed, the silicone was poured into the muffle. After the setting time, the natural teeth were extracted from the silicone. An epoxy resin for impregnation (Hardrock 554 “Batch n. 52245” + Hardener 554C “Batch n. 20160419”, Remet, Bologna, Italy) with a high dimensional stability was used to obtain the replicas. The resin/catalyzer ratio used was 100:20. After mixing slowly and continuously with a glass spatula to avoid bubbles, resin was poured into the cavities obtained in silicone. Complete hardening was obtained in 24 h at a temperature of 23 °C.

### 2.4. Bonding of Brackets

The labial surfaces of enamel were cleaned with a rubber cup with pumice and water for 10 s, washed for 20 s, and dried with oil-free air for 10 s with an air syringe. Subsequently, 36% gel orthophosphoric acid was placed on the buccal surfaces of enamel (DeTrey Conditioner 36, Dentsply DeTrey GmbH, Konstanz, Germany). Etchant was left to act for 20 s, after which it was rinsed with water and dried for 20 s, until the enamel’s surface took on a chalky white appearance. After etching, adhesive was applied (Ortho Solo, Ormco Corporation, Glendora, CA, USA) and positioned on the enamel with a microbrush. A small amount of composite resin (Grengloo, Ormco Corporation, Glendora, CA, USA) was placed on the base of brackets (Victory; 3M UniteK, Monrovia, CA, USA), which were then placed on the surface of teeth by exerting a slight pressure. After removal of the excess composite resin with a dental probe, each sample was light-cured for 40 s (20 s from the mesial side and 20 s from the distal side) with a lamp reaching an emission of 1600 mW/cm^2^ (Ortholux™ Luminous curing lamp, 3M Unitek, Monrovia, CA, USA). The light curing unit effectiveness was assessed every time before use. Specimens were stored in distilled water at 37 °C for 24 h and then underwent 30,000 thermal cycles in deionized water from 5 to 55 °C (LTC100; LAM Technologies Electronic Equipment, Firenze, Italy) [8].

### 2.5. Debonding and Clean-Up

Brackets were then removed by gently pressing the mesial and distal wings using a straight orthodontic clip (ETM 800-1001, ATS Plier GmbH & Co. KG, Hasbergen, Germany) to break the links at the bracket–adhesive interface.

Samples were randomly divided into two equal groups. In the first group, all of the finishing and polishing procedures were performed without using magnifying systems. In contrast, for the samples of the second group, all of the clean-up procedures were performed under constant observation using an operating microscope at a magnification of 10X (SOM32, Karl Kaps GmbH & Co. KG, Wetzlar, Germany).

Then, each group was further subdivided into four equal subgroups, in order to compare the different techniques used for clean-up (SO: Sof-Lex, EP: Enhance-PoGo, RU: single-pass rubber, and BLD: blade). Each subgroup was formed of five natural teeth with the respective pre-bonding replicas. For all subgroups, the first phase of composite residue removal was carried out with a multi-blade tungsten carbide cutter (H22 Algk.204.016, Komet Dental, Lemgo, Germany) mounted on a contra-angle handpiece (red ring). The subsequent phases were performed as follows.

#### 2.5.1. SO (Sof-Lex)

Sof-Lex discs were used for the finishing phases (Sof-Lex, medium, fine, and superfine, Ø 12.7 mm-1980, 3M ESPE AG, Seefeld, Germany) with decreasing granulometry mounted on a contra-angle handpiece. Disks were used with continuous movement, in order to avoid creating grooves in the enamel and with interrupted cooling via water spray. 

#### 2.5.2. EP (Enhance-PoGo)

The finishing steps were performed using Enhance finishing disks (Enhance Disks for finishing, Code: 624045, Dentsply Sirona, Milford, DE, USA) consisting of polymerized urethane-methacrylate resin, aluminum oxide, and silicon dioxide, followed by PoGo polishing disks (Enhance PoGo Tip Refill Discs, Code: 662010, Dentsply Sirona, Charlotte, NC, USA) consisting of polymerized urethane-methacrylate resin, fine diamond powder, and silicon dioxide. Both the finishing and polishing disks were mounted on a conventional handpiece and used according to the manufacturer’s instructions. 

#### 2.5.3. RU (Single-Pass Rubber)

Finishing was performed using a soft single-pass rubber impregnated with diamond grains (9524.UF.204.050, Komet Dental, Lemgo, Germany) mounted on a contra-angle handpiece and used at a speed between 6000 and 15,000 rpm with water spray cooling, as indicated by the manufacturer.

#### 2.5.4. BLD (Blade)

Clean-up was performed using a scalpel handle with a no. 15 blade (Code: 10-255-15, Hu-Friedy Mfg. Co., Frankfurt, Germany). The blade was used with an inclination of 45° on the tooth’s surface with corono-apical and mesio-distal movements and a spray of air was used during the finishing to remove the residue.

All of the techniques described above were performed by the same clinical operator, and the execution times of the finishing and polishing phases of the groups under examination were measured from the moment the instrument was placed on the tooth’s surface to the end of the procedures.

After the clean-up, natural tooth samples were again replicated using the same technique employed for the pre-bonding samples.

### 2.6. Observation with an Operative Stereomicroscope

The first morphological observation was performed by means of a stereoscopic microscope with 10× magnification (SOM32, Karl Kaps GmbH & Co., Wetzlar, Germany), which was operated by a single operator in order to evaluate the following:

- The fidelity of the replicas, comparing natural teeth post-clean-up with the respective replicas.
- The enamel damage index (EDI), introduced in 1990 by Howell and Weekes [9], which can take four values on a numerical scale from 0 to 3:

0 = Smooth surface without scratches. Perikymata are visible;
1 = Acceptable surface with only a few superficial scratches;
2 = Numerous deeper scratches and grooves;
3 = Scratches and grooves are visible to the naked eye.

### 2.7. Scanning Electron Microscope (SEM) Analysis

**Figure 1:**
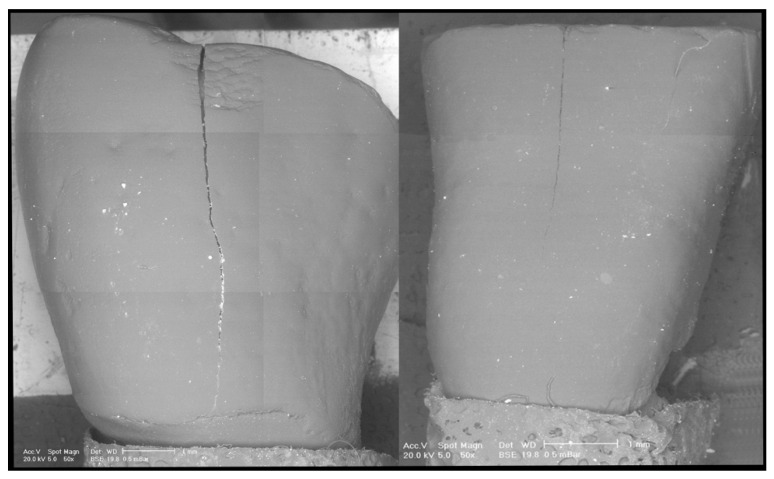
Representative scanning electron microscope (SEM) microphotograph of two replica samples before treatment.

**Figure 2:**
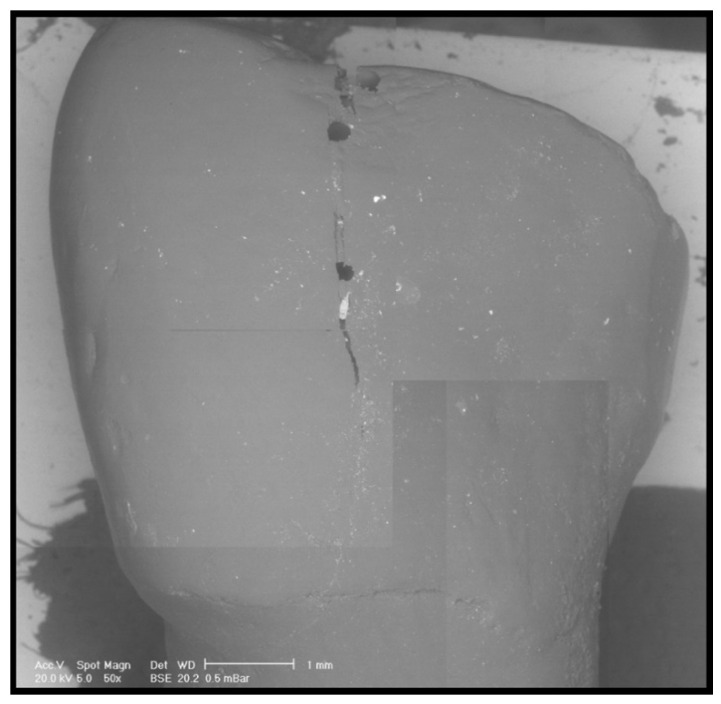
Representative SEM microphotograph of a replica belonging to the group treated with the aid of an operative microscope.

**Figure 3:**
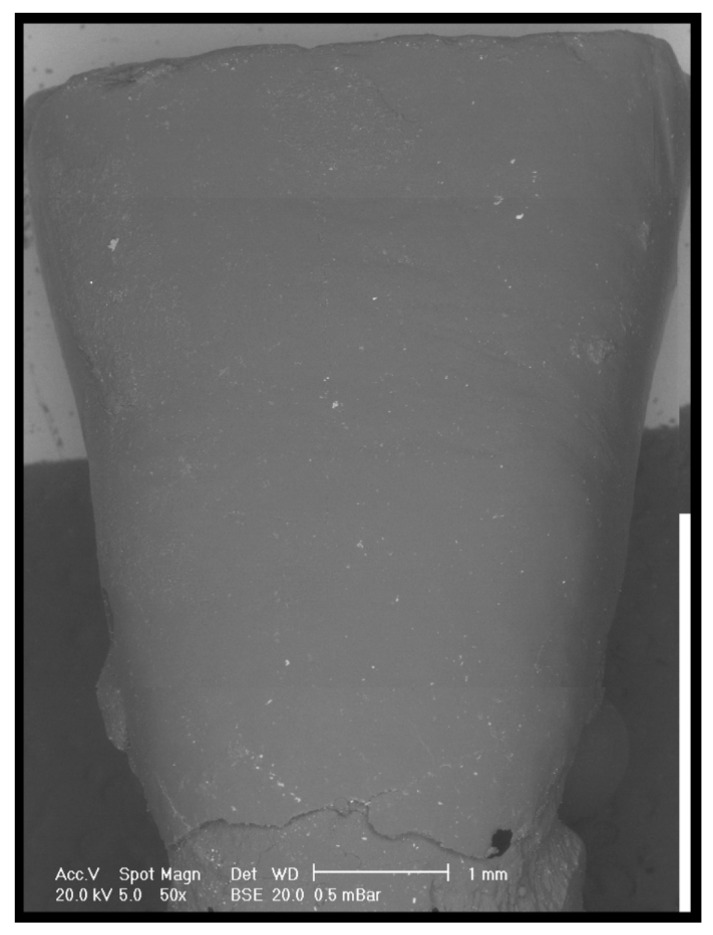
Representative SEM microphotograph of a replica belonging to the group treated without the aid of an operative microscope.

Pre- and post-clean-up replicas were dried with compressed air, mounted on an aluminum platform, and analysed under controlled pressure with a scanning electron microscope (SEM), as previously described [10,11,12,13,14] (XL 30 CP, Philips, Eindhoven, The Netherlands). SEM observations and comparisons of the pre-treatment images and the post-treatment images allowed us to distinguish the residual adhesive (Figure 1, Figure 2 and Figure 3). In addition, a quantitative analysis was performed by means of Image J (National Institutes of Health, Rockville Pike, Bethesda, MD, USA). The thresholding was chosen based on the observation of the SEM picture. Once those elements associated with the residuals remained on the screen, the threshold value was selected.

**Figure 4:**
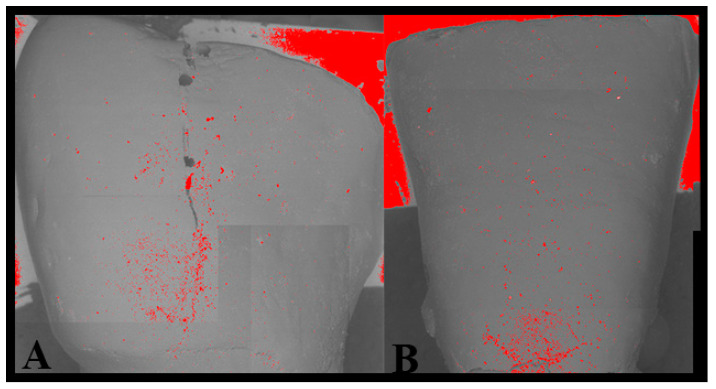
Segmentation of the SEM images. The red particles represent the area occupied by the adhesive residuals. (**A**) Sample debonded with the aid of the microscope. (**B**) Sample debonded without the aid of the microscope.

The adhesive remnant index [15] (ARI), which measures the amount of residual adhesive on the enamel, was adopted to compare specimens (Figure 4). 

This index, introduced by Artur and Bergland in 1984, measures the amount of composite residue on the enamel after debonding and has been modified in this study to be used after clean-up. The ARI can take four values, both in original and modified forms, on a numerical scale from 0 to 3, as follows:

0 = No adhesive on the enamel (0%);
1 = Percentage of residual adhesive on the enamel between 1% and 10%;
2 = Percentage of residual adhesive on the enamel between 11% and 20%;
3 = Percentage of residual adhesive on the enamel > 20%.

### 2.8. Statistical Analysis

All statistical analyses were performed using R software (v 3.5.1) (R Foundation for Statistical Computing, Vienna, Austria).

Continuous measures (ARI and the operative time) were analyzed by ANOVA and Tukey’s HSD for group comparisons, while the categorical variable (EDI) was evaluated by the chi-squared test. The significance level was set at *p* < 0.05. The normality of the data was tested using the Shapiro–Wilk test. 

## 3. Results

**Figure 5:**
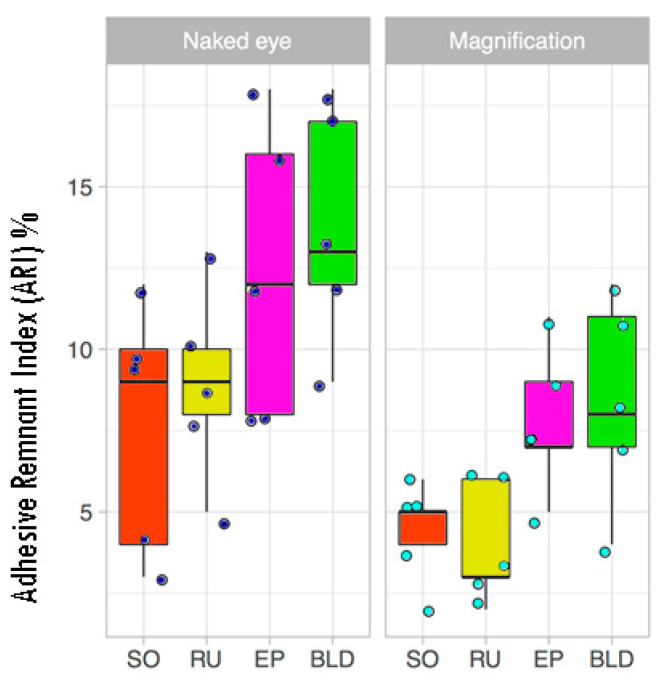
Boxplot of the resin residuals (adhesive remnant index, ARI). In addition, dots represent single percentage scores.

The null hypotheses tested were both rejected. Overall, magnification improved the removal of resin compared to the naked eye (*p* < 0.001) (Figure 5). The use of magnification constantly reduced the residual resin; specifically, SO and RU were the best techniques, while BLD was the worst technique for the ARI parameter. The same trend was also observed in the naked eye group (see Figure 5 for details). In the magnification group, the following significant differences were recorded: EP vs. SO (*p* = 0.024), BLD vs. SO (*p* = 0.003), and BLD vs. RU (*p* = 0.001). No significant differences were recorded between subgroups in the naked eye group. 

Even in terms of the surface damage (EDI), the use of the operative microscope in the microscope group reduced the EDI values when compared to the naked eye group (*p* = 0.0080) (11 vs. 3 “2 values”, respectively, in the naked eye and magnification groups). No 0 or 3 values were recorded in any group. Overall, no differences were observed within and between groups in terms of clean-up techniques (Table 1).

Conversely, it is relevant to consider that the use of the operative microscope in the microscope group significantly increased the operative times compared with the naked eye group (170 s/tooth vs. 70 s/tooth). No differences in terms of time were observed when comparing the different techniques tested.

## 4. Discussion

In this study, the effectiveness of four clean-up techniques, performed with or without the use of an operating microscope, were assessed in qualitative and quantitative terms. The results of the study highlighted two fundamental principles: enamel damage and residual adhesive after clean-up procedures still represent an ascertained risk in orthodontics, and the use of a magnification system improves the quality of debonding and clean-up procedures in a significant way.

The clinical procedure of bracket removal and finishing/polishing of the enamel covered by residual resin have been proved to be “potentially harmful”, as reported in other studies [16,17,18]. In this study, four enamel finishing techniques were compared. These were all preceded by a phase of the removal of composite residues, which has been proved to be effective in removing coarse residual resin. The comparison of the four finishing techniques showed that the SO and RU techniques displayed similar results for both the ARI and EDI. Today, the application of Sof-Lex discs is one of the most used techniques for finishing enamel [7]. In this study, SO discs mounted on contra-angle handpieces were used with a decreasing particle size. Discs were not used on the same working point for too long a period, in order to avoid creating grooves in the enamel.

The use of rubber impregnated with diamond grains (RU) showed low scores, which is probably due to the fact that the polishing system was developed to be used on composite materials and not on enamel and is therefore is more conservative [19]; the finishing phase carried out using Enhance and PoGo finishing rubbers (EP) resulted in damage to the enamel (in terms of scratches and grooves), which is probably due to the Enhance rubber that can become extremely abrasive if used with excessive pressure and on the same working point. 

Ulusoy reported that the effect of one-step and multi-step polishing systems on residual resin removal from enamel was dependent on the characteristics of the instrument in each system [20].

In the group finished with a scalpel (BL), the EDI was found to be 2 for all four samples. Moreover, there were diagonal and vertical scratches (more or less deep) and grooves in all four samples due to the lack of precision of this technique, especially without magnification.

Comparisons of the results of the four techniques used showed that regardless of the tools and finishing technique chosen, the use of a magnification system greatly influenced the results, improving both the ARI and EDI. This data is confirmed in several studies, including that of Baumann et al. 2011, which suggested the use of dental loupes during the debonding and clean-up phases to reduce the damage to enamel caused by these procedures [21]. 

Magnification systems are used daily in dental practice, especially in endodontics and microsurgery interventions [22,23]. The advantages of using a magnification system include the amplification of the smallest details, greater versatility in image magnification, visualization of the working field, better lighting, and a better working posture. The magnification of the smallest details is an especially important factor to consider during the debonding procedure, in order to save as much enamel tissue as possible during adhesive removal [24,25,26]. 

The literature reports several methods for investigating the surfaces of enamel [19,23,24,25,26,27,28]. As a result of the quantitative data provided, atomic force microscopy, environmental SEM, a profilometer, energy dispersive X-ray spectroscopy, and in vivo confocal microscopy analysis were used for these studies [29,30,31]. In particular, atomic force microscopy allows the morphological and mechanical properties of dental tissue surfaces with no peculiar treatment to be investigated [32]. However, within its limits, SEM was shown to be a good investigative tool for the observation of residual adhesive and enamel damage [4,33]. In the present study, in addition to the SEM observation employed to evaluate the surfaces of enamel, the image quantitative analysis approach was used. Image quantitative analysis is a good support for withdrawing quantitative data from histological and electron microscopy images, within the limit of these techniques [34,35].

In 2017, Bijelić et al. used open source software to segment histological images, in order to quantify the growth plate and trabecular bone in a mouse model [36].

FIJI thresholding is considered to be one of the best segmentation algorithms in crack image analysis [37] and is therefore very suitable for the analysis of enamel surface damage.

In the present study, this type of analysis, which represents the originality of the present work, allowed us to quantitatively confirm the qualitative observations of the operator, making it possible to recognize the area of damaged enamel, measure the true treated area, and calculate the percentages of residual adhesive. 

The results of this study confirm that none of the tested clean-up techniques can lead to complete atraumatic debonding. Not all of the techniques used for finishing and polishing the enamel produce similar results; the use of magnification appeared particularly significant. It led to less surface damage and less residual adhesive compared to the same techniques performed without magnification.

## Figures and Tables

**Table 1:** Pairwise comparisons of the enamel damage index (EDI) results using chi-squared tests. The reported values represent the *p* values obtained.

Naked Eye				Magnification			
	BLD	EP	RU		BLD	EP	RU
**EP**	1.00			**EP**	1.00		
**RU**	0.82	1.00		**RU**	0.84	1.00	
**SO**	0.40	1.00	0.82	**SO**	0.59	0.35	0.72
